# Interpretation of Fiber Supplementation on Offspring Testicular Development in a Pregnant Sow Model from a Proteomics Perspective

**DOI:** 10.3390/ijms20184549

**Published:** 2019-09-13

**Authors:** Yan Lin, Lujie Li, Yang Li, Ke Wang, Dongqin Wei, Shengyu Xu, Bin Feng, Lianqiang Che, Zhengfeng Fang, Jian Li, Yong Zhuo, De Wu

**Affiliations:** 1 Key Laboratory of Animal Disease-Resistance Nutrition and Feed Science, Ministry of Agriculture, Sichuan Agricultural University, Chengdu 611130, China; 18428387980@163.com (L.L.); lyang318@163.com (Y.L.); shengyuxu@sicau.edu.cn (S.X.); fengbin@sicau.edu.cn (B.F.); zhuoyong@sicau.edu.cn (Y.Z.); 2 Key Laboratory of Animal Disease-Resistance Nutrition, Ministry of Education, Wenjiang 611130, China

**Keywords:** fiber, maternal, proteomics, testis development

## Abstract

To study the effects of maternal fiber supplementation during pregnancy on the testicular development of male offspring and its possible mechanisms, 36 sows (Landrace × Yorkshire) were allocated to either a control diet (*n* = 18) or a fiber diet (the control diet supplemented with 22.60 g/kg inulin and 181.60 g/kg cellulosic; *n* = 18) during pregnancy. The body and testes weight of the offspring, 7-day-old piglets, was recorded. Testes were collected for further analyses. Results showed that the testicular organ index and the number of spermatogonia in single seminiferous tubule were higher in piglets from the fiber group than from the control group (*p* < 0.05). In addition, a significant increase in the concentration of glucose, lactate, and lipids in the testes was found in the fiber group (*p* < 0.05). Proteomic analysis suggested that there were notable differences in glucolipid transport and metabolism, oxidation, and male reproduction-related proteins expression between the two groups (*p* < 0.05). Results revealed that the most enriched signaling pathways in the fiber group testes included starch and sucrose metabolism, fatty acid metabolism, glutathione metabolism, and the renin-angiotensin system. mRNA expression analyzes further confirmed the importance of some signaling pathways in maternal fiber nutrition regulating offspring testicular development. Our results shed new light on the underlying molecular mechanisms of maternal fiber nutrition on offspring testicular development and provided a valuable insight for future explorations of the effect of maternal fiber nutrition on man reproduction.

## 1. Introduction

Studies on humans [1] and animals [2,3,4,5] show that pregnancy is the key window of testicular development. During pregnancy, the male fetuses’ seminiferous tubules gradually form, Leydig cells develop within the testicular interstitial tissue [6], both Sertoli cells and Leydig cells are abundantly produced [2,4,5], and primordial germ cells gradually differentiate into spermatogonia [7]. There is evidence showing that Leydig cells have a steroidogenic function, which maintains sex differentiation and promotes reproductive organ development [6]. Sertoli cells nourish spermatogonia [8] during spermatogenesis. The number of Sertoli cells and spermatogonia formed during embryogenesis is highly correlated with both adult testicular size and sperm production [9], while the embryonic period is the most active proliferation period of these cells [8]. Studies shows that poor maternal nutrition during gestation decreases the number of Sertoli cells present in newborn lambs [10]. Furthermore, both protein and energy restrictions in rats during gestation decreased offspring body weight, testicular weight, and estradiol body content, while increased testosterone concentration in the testicles [11,12]. Additionally, protein restriction during pregnancy reduces growth hormone levels in sows offspring [13], and causes testicular growth retardation in rat offspring as well [14]. It has also been seen that testicular dysplasia in male lambs is caused by energy restriction during ewes’ pregnancy [15]. A study using hamsters also revealed that maternal nutritional restriction affects reproductive organ size, hormone levels, and the development of the testes and the epididymis in the offspring [16]. Undernutrition during early fetal development was clearly associated with an increased expression of the steroidogenic acute regulatory protein [17]; an excessive steroid production may adversely affect fetal organ development. In addition, pregnancy conditions may affect the future development of the reproductive organs and the adults’ reproductive level of male offspring [18,19]. These implicates that maternal nutrition during pregnancy has far-reaching significance on testicular development and spermatogenesis of the offspring even after adulthood.

Fiber is a significant nutrient. Recent research data show that fibers play an important role in health and reproduction, both in humans [20,21,22,23] and animals [24,25,26]. Fibers can improve glucose and lipid metabolisms, and enhance the antioxidation ability of both mice and humans [22,27]. In addition, it has been seen that, when fiber is regularly included in the diet, it may improve the development of the female reproductive organs [28], leading to increased female fecundity.

Fiber supplementation also affects testes development of young mice [25]. In men, increasing fiber intake increments the levels of the sex hormone-binding globulin that affects the activity of related sex hormones [20] which, in turn, regulate testicular development. Other studies found that feeding sows with a high fiber diet during pregnancy would improve their reproductive performance as well as immunity and birth weight of newborn piglets [24]. Increasing maternal fiber intake during pregnancy could also reduce the risk of breast cancer in offspring [21] and affect glucolipid metabolism and antioxidant capacity of piglets [29,30].

Nevertheless, it is not clear whether maternal fiber supplementation during pregnancy affects testicular development of offspring and, if so, what are its mechanisms. Thus, we used a sow model and proteomics techniques to study the effects of maternal fibrous nutrition during pregnancy on testicular development of offspring. This work demonstrates that maternal fiber intake during gestation has great effects on testicular development, and revealed that glucolipid metabolism, glutathione metabolism, and the renin-angiotensin system mediated the effect of maternal fiber nutrition over offspring testicular development.

## 2. Results

### 2.1. Effect of Maternal Fiber Intake on Body Weight and Testicular Development of Offspring

**Figure 1:**
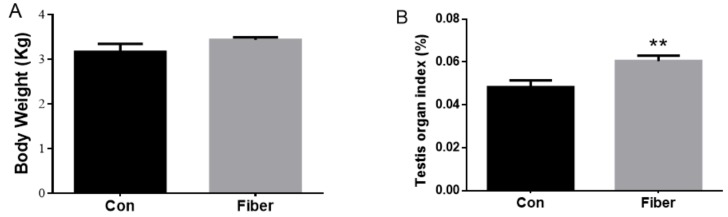
Effect of maternal fiber intake on the body weight (**A**) of offspring, (**B**) testis organ index of offspring. The values were expressed as mean ± SEM. ** *p* < 0.01, as compared to normal control group. Con = control group, Fiber = Fiber group.

**Figure 2:**
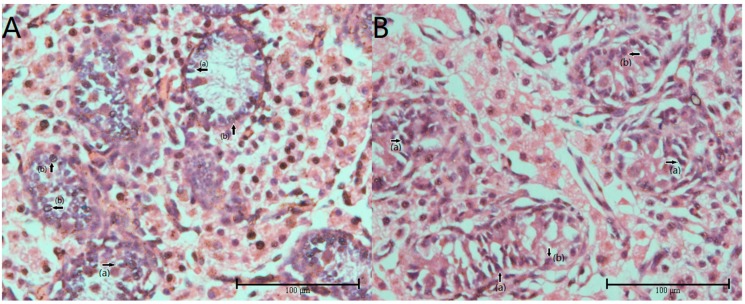
Testis tissue cross-sections of piglets, stained in hematoxylin-eosin. (**A**) testis of piglets in control group, 400×. (**B**) Testis of piglets in fiber group, 400×. (a) Stood for Sertoli cell and (b) stood for spermatogonia.

**Figure 3:**
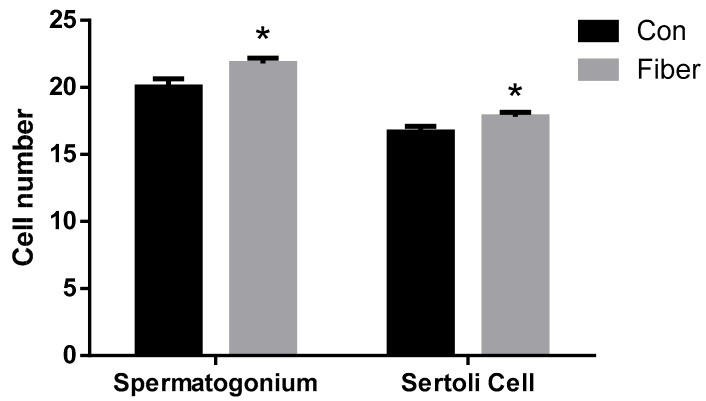
Effect of maternal fiber intake on the number of spermatogonium and Sertoli cells at per seminiferous tubule in testes of offspring. The values were expressed as mean ± SEM. * *p* < 0.05, as compared to normal control group. Con = control group, Fiber = Fiber group.

There were no significant differences in body weight of 7-day-old piglets from the two groups at (*p* > 0.05; Figure 1A), whereas, in the fiber group (hereafter Fiber), the testicular organ index was significantly higher than in the control group (Con) (*p* < 0.001; Figure 1B). Figure 2 shows the testicular tissue from Con Figure 2A and Fiber Figure 2B 7-day-old piglets, showing Sertoli cells (a) and spermatogonia (b). Both the number of spermatogonia and Sertoli cells per seminiferous tubule were significantly higher in Fiber than in Con testes (*p* < 0.05; Figure 3).

### 2.2. Effect of Maternal Fiber Intake on Offspring Biochemical Parameters

**Figure 4:**
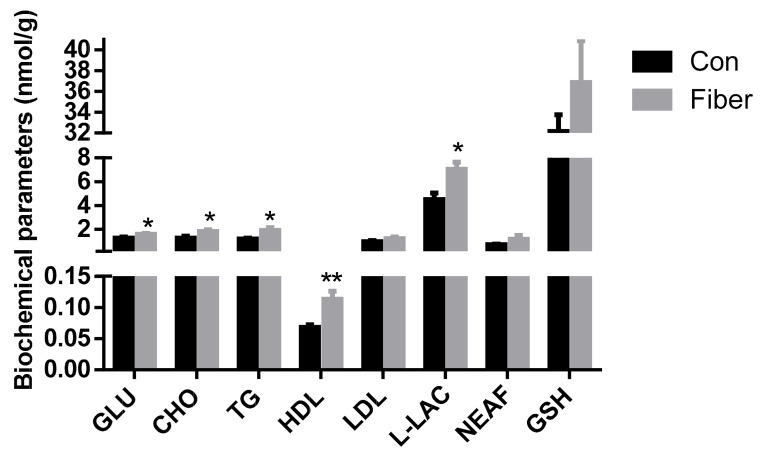
Effect of maternal fiber intake on the biochemical parameters of offspring. The values were expressed as mean ± SEM. * means *p* < 0.05, ** *p* < 0.01, as compared to normal control group. Con = control group, Fiber = Fiber group. GLU = glucose, CHO = cholesterol, TG = triglyceride, HDL = high density lipoprotein, LDL = low density lipoprotein, l-LAC = l-lactate, NEFA = non-esterified fatty acid and GSH = l-Glutathione.

Regarding biochemical compounds, compared with Con testes, the concentration of triglycerides (TG), cholesterol (CHO), high density lipoproteins (HDL), glucose (GLU), and l-lactate (l-LAC) in Fiber testes was significantly higher (*p* < 0.05), whereas the concentration of low density lipoproteins (LDL), non-esterified fatty acids (NEFA), and l-glutathione (GSH) showed no significant difference (*p* > 0.05; Figure 4).

### 2.3. DEPs Analysis in Proteomics

We identified 6481 proteins, among which, 5718 proteins contained quantitative information. We established a 1.2 times change as the change threshold and determined that 25 proteins were upregulated whereas 24 were downregulated in Fiber compared to Con. Among them, there were 6 proteins related to male reproduction, 5 to carbohydrate metabolism, 2 to fatty acid metabolism, and 2 to glutathione metabolism (Table 1). The complete information on differentially expressed proteins (DEPs) is shown in Table S1.

### 2.4. Functional Enrichment Analysis of DEPs

**Figure 5:**
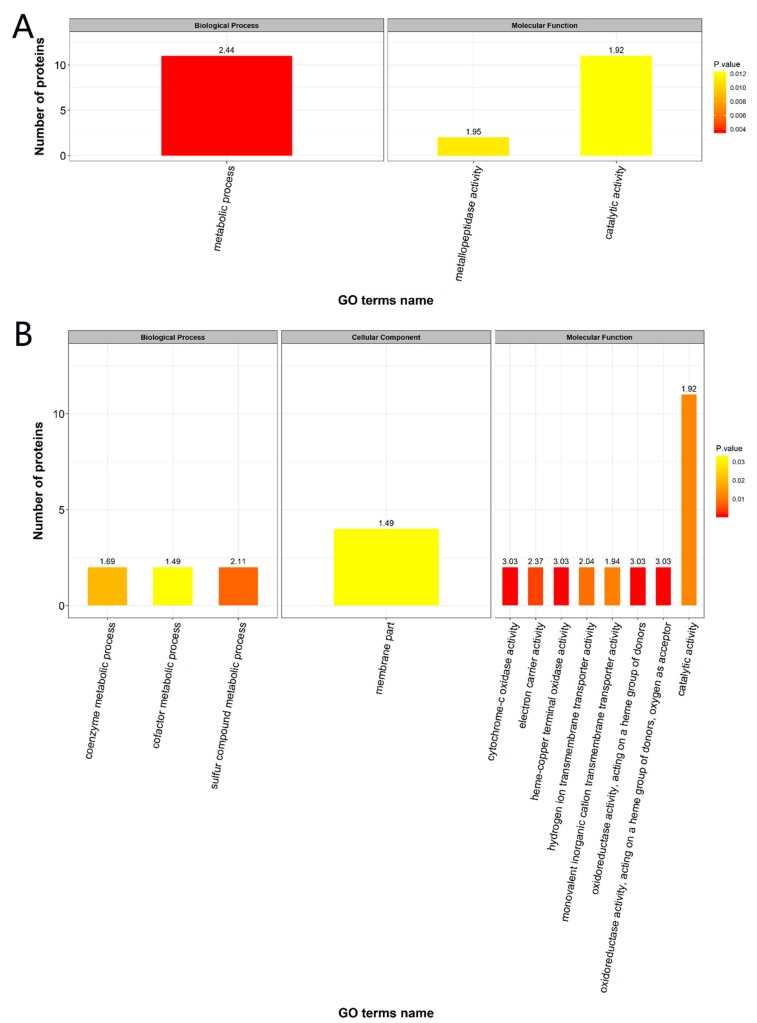
The functional enrichment of Gene ontology (GO) annotation of the up-regulated DEPs (**A**) and down-regulated DEPs (**B**). The number on the histogram represents the negative logarithmic transformation of the *p*-value obtained by the enrichment test (using Fisher’s exact test).

Gene Ontology (GO) terms annotation showed that upregulated DEPs were enriched in biological process (BP) and molecular function (MF; Figure 5A). 24 upregulated DEPs were able to be annotated according to GO information. The complete information on DEPS is shown in Table S2. BP included metabolic processes, whereas MF included metallopeptidase activity and catalytic activity; the upregulated proteins had their highest enrichment in metabolic processes (Figure 5A). The downregulated proteins were enriched in BP, cellular component (CC) and MF (Figure 5B); there were 35 DEPs able to be annotated according to the GO database information. BP enrichment included the metabolism of sulfur compounds, cofactor and coenzyme, CC included membrane part, and MF was mainly enriched for enzyme activities. Cytochrome-c oxidase activity, heme-copper terminal oxidase activity, and oxidoreductase activity were the most significantly enriched GO terms in downregulated proteins, whilst catalytic activity was enriched for most proteins.

**Figure 6:**
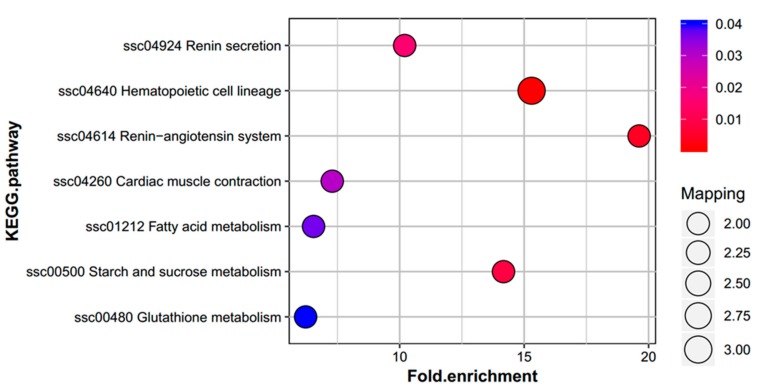
Kyoto Encyclopedia of Genes and Genomes (KEGG) pathway enrichment analysis of DEPs. There were 15 DEPs were able to be enrichment. Hematopoietic cell lineage, renin-angiotensin system and starch and sucrose metabolic pathways were most significantly enriched.

Furthermore, a KEGG pathway enrichment analysis showed 15 DEPs involved in renin secretion, hematopoietic cell lineage, the renin-angiotensin system, cardiac muscle contraction, fatty acid metabolism, starch and sucrose metabolism, and glutathione metabolism (Figure 6). All KEGG pathways information is shown in Table S3. Hematopoietic cell lineage, the renin-angiotensin system, and starch and sucrose metabolic pathways were the most significantly enriched pathways, form these, the hematopoietic cell lineage pathway was enriched with the highest number of proteins. DEPs in the glutathione metabolic pathway included F1RLR8 and P36968; in starch and sucrose metabolism pathway, A0A286ZUF1 and F1RQQ8; in the fatty acid metabolism pathway, I3LP02 and K7N7E5; and in the renin-angiotensin system pathway, F1RRW5 and A0A287B315.

### 2.5. Relative Expression of mRNAs

**Figure 7:**
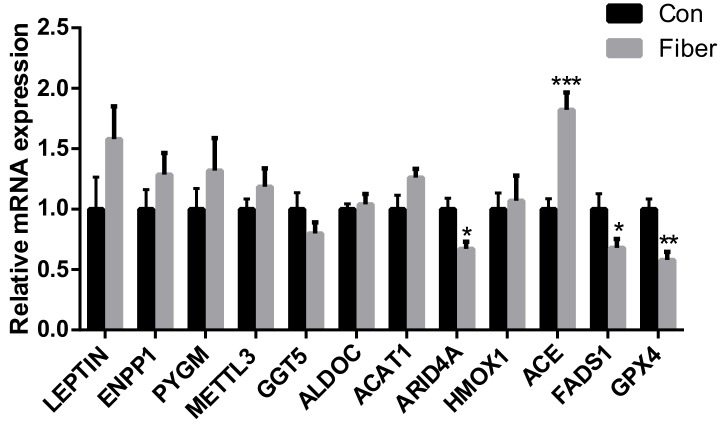
Effect of maternal fiber intake on the relative expression of mRNA. The values were expressed as mean ± SEM. * means *p* < 0.05, ** *p* < 0.01, *** *p* < 0.001, as compared to normal control group. Con = control group, Fiber = Fiber group.

Compared to the Con, the relative expression of mRNAs of the fatty acid metabolism (e.g., *FADS1*), male reproduction (e.g., *ARID4A*), and the renin-angiotensin system (e.g., *ACE*) proteins was significantly decreased in Fiber (*p* < 0.05; Figure 7); whereas the relative mRNA expression of glutathione metabolism (e.g., *GPX4*) gene was significantly increased (*p* < 0.01; Figure 7). In addition, there was no significant difference in the relative mRNA expression of fatty acid metabolism (e.g., *ACAT1*), glutathione metabolism (e.g., *GGT5*), Carbohydrate metabolism (e.g., *ENPP1*, *PYGM,* and *ALDOC*) and the male reproductive (e.g., *HMOX1*, *LEPTIN,* and *METTL3*) (*p* > 0.05; Figure 7).

## 3. Discussion

Results showed that fiber supplementation of sows during pregnancy was beneficial for male piglets, inducing an early development of the testes and an increase in the testicular organ index; the higher the testicular organ index, the better the nutritional environment for testicular development [9]. To our knowledge, this is the first study that shows that the supplementation of the maternal diet with fiber during pregnancy could increase the number of Sertoli cells and spermatogonia in offspring testes. The more spermatogonia and Sertoli cells, the more conducive to testicular development, which may have long-term effects in offspring reproductive performance.

GO analysis and KEGG pathway analysis of our data showed that many of the DEPs are involved in carbohydrate metabolism, fatty acid metabolism, GSH metabolism, and renin-angiotensin system pathways. These DEPs may play an important role in early testicular development; related pathways may also be involved in regulating the early development of testes. Now, it has been demonstrated that carbohydrate metabolism plays an important role in nutrient supply to testes by Sertoli cells [31,32]. In this regard, *ENPP1*, *PYGM*, and *ALDOC* are all involved in carbohydrate metabolism. Although there were no previous studies about their role in male reproduction, we found that the GLU content in offspring testes of Fiber was 26.43% higher than that of Con, suggesting that maternal fiber intake may alter testicular GLU supply to the offspring. It is well-known that GLU is one of the main substances that pass through the placental barrier [33]. After glucose enters the fetus, mediated by glucose transporters (GLUTs), it is metabolized and transmitted to the testes, diffusing through the blood-testis barrier. In the testis, Sertoli cells use GLU to produce lactate [31]. Neither spermatocytes nor sperm cells can use GLU directly, but utilize lactate as an energy source [32]. Lactic acid increases protein synthesis by producing adenosine triphosphate (ATP); then, it regulates the rate of nicotinamide adenine dinucleotide (NADPH) oxidase oxidation and the pentose phosphate pathway, and plays a role in other metabolic pathways in sperm cells [34]. In fact, the lactic acid concentration in the testis of Fiber was 55.96% higher than that in Con. Several studies have shown that infusion of lactic acid in testes can improve spermatogenesis in adult cryptorchidism [35], and that it has antiapoptotic effects on germ cells [34]. Therefore, the secretion of lactate by Sertoli cells is essential for spermatogonia. Sertoli cells ensure enough lactic acid supply in the microenvironment where germ cells develop, even in the absence of glucose [36]. This suggests that fiber intake by the mother may affect testicular development by impacting carbohydrate metabolism, thereby striking the energy supply of both glucose and lactate in the testis.

Besides, the process of testis development needs plenty of lipids [37]; fatty acid metabolism is important in early testis development in the fetus. *ACAT1* and *FADS1* genes are involved in fatty acid metabolism, and their expression affects CoA production, which in turn affects the β-oxidation process and fatty acid biosynthesis. *ACAT1* contributes to the balance of CHO in the body, essential in the process of sperm production [38]. The expression of *FADS1* is related to fatty acid synthesis, and CHO synthesis is positively correlated with *FADS1* gene expression [39]. The CHO, TG, and HDL content in offspring testes in Fiber were higher than those of Con, and the expression of *ACAT1* and *FADS1* was also higher in Fiber compared with Con, indicating that maternal fiber intake may alter offspring testicular lipid content. TGs do not cross the placental barrier directly, while lipoprotein receptors, lipoprotein lipase, phospholipase A2, and intracellular lipase are introduced into the fetus to resynthesize triglycerides [33,37]. In spermatogenic epithelial cells, Sertoli cells can synthesize CHO in vitro, but the CHO concentration required for steroid production or spermatogenesis exceeds the ability of Sertoli cells to synthesize the compound, thus, testicular CHO synthesis depends on lipid transport in the blood circulation [40]. Some studies have shown that Omega-3 polyunsaturated fatty acids have no effect on the survival of TM4 cells, but that they can improve the cell viability caused by palmitic acid [41]. Jutika Datar [42] found that the genes involved in triacylglycerol synthesis and sphingomyelin metabolism of phosphatidylglycerol were barely expressed in the dysplastic testes of obese rats as determined by transcriptome sequencing. Hermo et al. [43] also found inactivation of genes involved in lipid metabolism in mice affected spermatogenesis, suggesting that genes related to fatty acid metabolism in testes play a crucial role in testicular development. This demonstrates that fibers may affect testicular development by affecting lipid metabolism, directly modifying lipid supplies, such as CHO, TG, and HDL, in the testis.

Glutathione metabolism, involving genes like *GPX4* and *GGT5*, is also related to Sertoli cell metabolism, important for testis development [31]. The expression of *GPX4* affects the synthesis of GSH and oxidized l-glutathione (GSSG). *GGT5* is a key gene, mainly expressed in mammalian Leydig cells; increasing the expression of *GGT5* may alter local oxidation status and impair steroid production in the testicles [44]. We found that the relative expression of *GPX4* in Fiber was 72.41% lower than that of Con, while there were no significant differences in the GSH content between the two groups. These data may indicate that enzyme interactions for GSH synthesis and metabolism, resulting in little changes in GSH content. In contrast, studies have shown that overexpression of *GPX4* may cause a fertility decrease [45]. It is known that Sertoli cell metabolism highly depends on GSH and monocarboxylate transporters [31]. Sertoli cells, peritubular cells, pachytene spermatocytes round spermatids, and interstitial tissues contain high concentration of GSH, whereas Sertoli cells and peritubular cells also have high GSH-dependent enzyme activities [46]. The addition of GSH can improve the total motility and plasma membrane integrity of ram sperm after thawing [47], and it has an effect on maintaining sperm motility [47]. Moreover, Stradaioli [48] found that high GSH levels in the extender can reduce oxidative damage over surviving sperm during freezing and thawing. It was also found that when GSH was added to the thawing agent, the sperm fertilization capacity would increase in direct proportion to GSH dose [49]. Mata-Campuzano discovered that cryopreserved spermatozoa supplemented with GSH could improve the sperm mitochondrial activity, resulting in an increase in litter size after artificial insemination [50]. We suggest that dietary fibers improve testicular development by changing the expression of GSH synthesis and metabolism enzymes.

Some studies have suggested that there is a specific renin-angiotensin system in testes. Testes can synthesize angiotensin-converting enzyme (ACE) [51] which regulates angiotensin production; in turn, angiotensin II may play a role in spermatogenesis, keeping sperm motility and fertilization [52]. *ACE* is a key checkpoint in the renin-angiotensin system; restrictive expression of *ACE* hinders the development of Leydig cells and spermatozoa [53]. This work showed that the expression of *ACE* in Fiber was 82.10% higher than in Con, suggesting that dietary fibers may affect the renin-angiotensin system by increasing ACE. Thus, fibers may increase germ cell number in the testes through changes in the renin-angiotensin system, which promotes testicular development. In addition, *METTL3*, *ARID4A*, *HMOX1*, and *LEPROT* are involved in male reproductive related processes: *METTL3* regulates spermatogonial differentiation and meiosis initiation [54]. *ARID4A* mainly expresses in testicular Sertoli cells, supporting spermatogenesis and constructing the blood-testis barrier; an *ARID4A* gene deletion leads to phenotypic dysfunction of Sertoli cells, spermatogenic disorders, damages to the blood-testis barrier, and increased seminiferous tubule permeability [55]. *HMOX1* can regulate the levels of reactive oxygen species of human seminiferous tubules during cryopreservation, in addition, it has cytoprotective effect and can ease cisplatin-induced reproductive toxicity in male rats. Additionally, some studies reveled that increasing the expression of leptin and leptin receptors (such as *LEPROT*) were inversely correlated with the secretion of testosterone (T), the main androgen formed by the fetal testes at the time of male sexual differentiation [56]. Thus, leptin and leptin receptors are important in testis development. As their expression is changed by maternal fibers intake, these changes may affect the testes development at some extent.

## 4. Materials and Methods

All experimental procedures followed the current law regarding animal protection and were approved by the Guide for the Care and Use of Laboratory Animals prepared by the Animal Care and Use Committee of Sichuan Agricultural University (permit number DKYB20131704, date 2 January 2018). The experiment was carried out at the Sichuan Agricultural University experiment teaching base.

### 4.1. Animals and Diets

A total of 36 gilts (Landrace × Yorkshire), 9-month-old each, were used in a 118-day trial. Sows began mating at the third estrus, and they were randomly divided into the control group (Con; *n* = 18) and the fiber group (Fiber; *n* = 18) when they were pregnant (randomized controlled trials registration number: ISRCTN50167738). Sows were fed with diet limitation during gestation to control their body weight. Diets were fed at 9 am every day. During the first 89 days of pregnancy, feeding levels were of 2.15 kg/day, whereas they were increased to 2.55 kg/day from day 90 to 110. Con was fed a corn-soybean basal diet according to the NRC (2012) (Table 2), whilst Fiber’s basal diet was supplemented with 22.60 g/kg inulin and 181.60 g/kg cellulosic (i.e., 48.59 g inulin and 390.44 g cellulosic during the first 89 days of pregnancy, and 57.63 g inulin and 463.08 g cellulosic from day 90 to 110). During the experiment, animals were housed in individual pens in a well-ventilated room with a temperature between 20 and 26 °C and controlled lighting for 12 h/day. Water was provided ad libitum. Daily cleaning and disinfection work were carried out to keep the room hygienic and dry. Sows were transferred to the farrowing room at day 110 of pregnancy. Sows were fed with a lactation diet (according to NRC 2012) after delivery. Cross-fostering was first allowed 24 h after farrowing and it was only carried out within each treatment group. Then, we recorded the individual weight of 7-day-old piglets.

### 4.2. Sample Collection

The testes of 7-day-old piglets were collected after anesthesia (Zoletil 50; Virbac, Nice, France) and weighted. Excised testes were dissected into two portions, one part was stored at −80 °C until analysis and the other part was fixed in 4% paraformaldehyde for 2 h.

### 4.3. Physiology and Biochemistry of Testes

Fixed testis tissues were embedded in paraffin and 5 µm sections were cut using a Leica microtome (Leica, Solms, Germany). Sections were then fixed in glass slides, stained with hematoxylin and eosin, and photographed under a light microscope (Motic, Xiamen, China) at a 100× magnification. We used Image-Pro plus 6.0 to count the number of seminiferous tubules, Sertoli cells, and spermatogonia per unit area to evaluate the testicular development [57]. For each section, 10 visual fields were selected; each visual field included at least 5 seminiferous tubules. After counting the number of seminiferous tubules and the cells within, the average number value was calculated for each cell type [10].

Some of the samples that were stored at −80 °C were thawed and homogenized in phosphate buffer (pH 7.4). Homogenates were centrifuged and the supernatants transferred to clean tubes. GSH content was measured using enzyme-linked immunosorbent assay kits (S0053, Beyotime, Shanghai, China) according to the manufacturer’s specification. The content of TG, CHO, LDL, HDL, NEFA, GLU, and l-LAC was measured using an automatic biochemistry analyzer (Hitachi 3100, HITACHI, Tokyo, Japan).

### 4.4. TMT Quantitative Proteomic

The testes of 3 piglets from each group were selected for proteomic analysis. Entrusting PTM Biolabs, Inc (Hangzhou, China) to achieve it. Briefly, each sample was grinded into powder in liquid nitrogen and mixed with a four times volume of lysis solution. Then, samples were subjected to supersonic splitting thrice. Samples were centrifuged and supernatants collected. The protein concentration per sample was determined using a BCA Protein Assay kit (P0011, Beyotime) according to the manufacturer’s specifications.

Dithiothreitol was added to each sample to a final protein concentration of 5 mM for reducing the protein’s disulfide bonds. After that, iodoacetamide was added to a final concentration of 11 mM and samples were incubated. The urea concentration in protein samples was diluted by adding 100 mM TEAB. Then, trypsin was added at a 1:50 trypsin-to-protein mass ratio for the first digestion and left stand overnight before adding trypsin once more to a 1:100 trypsin-to-protein mass ratio for a second 4 h digestion period.

Peptides were desalted using a Strata X C18 SPE column (Phenomenex, Los Angeles, CA, USA) and vacuum dried. We reconstituted peptides in 0.5 M TEAB and processed them according to the instructions of the TMT kit (Scientific™, Thermo, Waltham, MA, USA).

Tryptic peptides were fractionated by high pH reverse-phase HPLC using an Agilent 300Extend-C18 chromatographic column (5 μm particles, 4.6 mm ID, 250 mm length).

Tryptic peptides were dissolved in an aqueous solution containing 0.1% formic acid and 2% acetonitrile (mobile phase A) and separated by the EASY-nLC 1000 system (Thermo Scientific). An aqueous solution of 0.1% formic acid and 90% acetonitrile was used as mobile phase B.

Our second-order mass spectrum data were retrieved by MaxQuant (v.1.5.2.8; Munich, Germany) using the following parameters: Trypsin/P as stablished as the digestion mode. The precursor ion mass errors of the first search and the main search were 20 and 5 ppm, respectively. The error for the fragment ions was 0.02 Da. Cysteine alkylation was set as a fixed modification, while the oxidation of Met and the acetylation of the N-terminus of the protein were set as variable modifications.

### 4.5. Bioinformatics Analysis

#### 4.5.1. Annotation Methods

Gene Ontology (GO) annotations at the protein level were derived from the UniProt-GOA database (www.http://www.ebi.ac.uk/GOA/; Hinxton, UK). First, identified protein IDs were converted to their corresponding UniProt IDs and then to their GO IDs. Based on these Go IDs, we retrieved the corresponding information from the UniProt-GOA database. When the identified proteins were not annotated by the UniProt-GOA database, we used InterProScan (Hinxton) to annotate the corresponding GO function of the proteins, based on a protein sequence alignment method. Then, proteins were classified by GO annotation into three categories: biological process (BP), cellular component (CC) and molecular function (MF).

Next, the Kyoto Encyclopedia of Genes and Genomes (KEGG) database was used to annotate those protein pathways in which relevant identified proteins participate. Using KEGG online service tools KAAS and KEGG mapper, we annotated submitted proteins, and mapped them on the KEGG pathway database, respectively.

#### 4.5.2. Functional Enrichment

GO annotation classifies proteins into three categories: biological process, cellular compartment, and molecular function. For each category, a Fisher’s exact test (two-tailed) was used to test the enrichment of the DEPs against the whole set of identified proteins. GO enrichment tests with a corrected *p*-value below 0.05 were considered significant.

In addition, the KEGG database was used to identify enriched pathways by a Fisher’s exact test (two-tailed) to test the enrichment of the DEPs against all identified proteins. Pathway enrichment tests with a corrected *p*-value below 0.05 were considered significant. Finally, these pathways were classified into hierarchical categories according to the KEGG website.

#### 4.5.3. Quantitative PCR Analysis

We selected specific parts of genes to check the results obtained from the proteomics analysis; the primers used are described in Table 3. We blended testicular samples with TRIzol to homogenization. Chloroform was added to each sample and the mix was shaken and then allowed to stand. Samples were centrifuged, the aqueous phase mixed with isopropanol and left stand still again. Samples were centrifuged, supernatants were discarded, and 75% ethanol was added to the pellets. Resuspension was done by continuous beating of the tubes. Once again, samples were centrifuged and RNA pellets were air dried and resuspended in DEPC water. RNA was reverse transcribed according to the reverse transcription kit’s instructions (Takara). For quantitative PCR, we used a real-time PCR system (Bio-Rad, Hercules, CA, USA) the amplification reaction was done according to the product specifications (Takara). β-actin was used as a reference gene; the relative expression of the target gene was calculated by the 2^−ΔΔ*C*t^ method [58].

### 4.6. Statistical Analysis

The data values of this study were expressed as mean ± SEM. All the data were analyzed by *t* test using SAS 9.4 software (Raleigh, NA, USA), *p* < 0.05 was considered as a statistically significant difference.

## 5. Conclusions

We identified 16 different DEPs which affect testis cell proliferation. Testis development in the offspring of sows with different fiber intake during gestation may be affected by the pathways to which these DEPs belong. The exerted effects on early testicle development may be due to the regulation of key points in carbohydrate metabolism, fatty acid metabolism, and GSH metabolism pathways as well as in the renin-angiotensin system. Our results improve our understanding of the molecular mechanisms associated to the effect of maternal fiber intake on offspring.

## Figures and Tables

**Table 1:** Differentially expressed proteins (DEPs) associated with Kyoto Encyclopedia of Genes and Genomes (KEGG) pathway in the fiber group and the control group of testes in piglets.

Protein Accession	Protein Description	Fiber/Con Ratio	Fiber/Con *p* Value	Gene Name
A0A287A1D6	Methyltransferase like 3	0.625	0.0488	*METTL3*
F1RJ25	Fructose-bisphosphate aldolase	0.757	0.025	*ALDOC*
P36968	Phospholipid hydroperoxide glutathione peroxidase	0.76	0.0039	*GPX4*
F1RLR8	Gamma-glutamyltransferase 5	0.802	0.0158	*GGT5*
K7N7E5	Uncharacterized protein	0.825	0.015	*FADS1*
F1SSK5	Uncharacterized protein	0.828	0.0164	*ARID4A*
I3LP02	Acetyl-CoA acetyltransferase 1	1.226	0.01	*ACAT1*
F1RQQ8	Alpha-1,4 glucan phosphorylase	1.247	0.0234	*PYGM*
B9TRX0	Leptin receptor gene-related protein	1.249	0.0378	*LEPROT*
F1RRW5	Angiotensin-converting enzyme	1.273	0.0286	*ACE*
A0A286ZUF1	Ectonucleotide pyrophosphatase/phosphodiesterase 1	1.321	0.00702	*ENPP1*
A0A287AWS9	Heme oxygenase	1.432	0.0239	*HMOX1*
A0A287BLE1	Sequestosome 1	1.216	0.0184	*SQSTM1*
F1SAZ0	Sperm associated antigen 17	0.351	0.0000791	*SPAG17*
A0A287A7G0	COX assembly mitochondrial protein	1.91	0.0139	*CMC2*
F1CNZ4	STEAP family member 4	1.318	0.0464	*STEAP4*

The change of protein expression level was expressed by the ratio of fiber/control group. The ratio >1 indicates up-regulation and the ratio <1 indicates.

**Table 2:** Control group diet and nutritive composition.

Material and Composition, %		Nutritive Composition	
Corn	62.39	DE, Mcal/kg	3.36
Peeled soybean meal	13.10	CP, %	13.39
Fish meal	2.00	CF, %	2.90
Flour	10.00	CF, %	1.41
Corn starch	10.00	Soluble fiber, %	1.13
Lys	0.10	Insoluble fiber, %	9.08
Thr	0.02	Insoluble/soluble	8.03
CaCO3	0.84	Dietary fiber, %	10.21
CaHCO3	0.46	Ca, %	0.60
NaCl	0.40	Available P, %	0.27
Choline	0.14	Lys, %	0.60
Sow multivitamin ^1^	0.05	Met, %	0.21
Mineral addition ^2^	0.50	Thr, %	0.46
Total	100.00	Trp, %	0.14

^1^ Multi-dimensional of breeding pig per kilogram: VA 17500IU, VD3 5000IU, VE 37.5IU, VK3 5 mg, VB1 5 mg, VB3 12.5 mg, VB6 7.5 mg, VB12 0.05 mg, biotin 0.2 mg, niacin 50 mg, folic acid 2.5 mg, D-calcium pantothenate 25 mg, ethoxyquinoline 0.25 mg. ^2^ Premix per kilogram: Cu 10 mg, Fe 100 mg, I 0.6 mg, Zn 100 mg, Mn 30 mg, Se 0.25 mg.

**Table 3:** Details of the primers.

Gene Name	Forward Sequence (5′→3′)	Reverse Sequence (5′→3′)	Accession No	Product Length (Base Pair)
*ACAT1*	GGCTTACCTATTTCTACTCCGTGC	CCATTCCACCTGCCACCAT	XM_005667301.3	127
*FADS1*	AGCCTTGCTGCCTGCCTACT	CAGTGGCACATAAGTGAGGAAGAT	NM_001113041.1	124
*GGT5*	AACACGGTTCACCTGTGGATG	CCTGTGGTCGCGTTGTAGATAGT	XM_021074378.1	118
*GPX4*	AACCAGTTTGGGAGGCAGGAG	GGACTTTCATCCACTTCCACAGAG	NM_214407.1	142
*PYGM*	ACGTGGACGACGAAGCCTTTA	TTGATGTGGACTTTGTATTCCCTCT	XM_003122588.5	103
*ALDOC*	GATAAAGGCATTGTCGTGGGC	GCAAAGTCGGCACCATCCT	XM_005656989.3	140
*METTL3*	CTTGCCCTTACACAGAGCGTTG	CAAACTTGCCCAAGATACTGACGT	XM_003128580.5	112
*ARID4A*	ATCTGCTCTTTCACCAAACATGC	TTCCATTCCATTTGACAGAGGTG	XM_021088206.1	124
*HMOX1*	CAGGCTGAGAATGCCGAGTT	CTTGTTGTGCTCAATCTCCTCCT	NM_001004027.1	129
*ACE*	ACGCCAACAGCACTTGTCTTC	ATGGCTCTGCCCACCTTGTC	NM_001033015.3	121
*LEPTIN*	TCATCAAGACGATTGTCACCAGG	TGGATCACATTTCTGGAAGGCA	XM_021078503.1	184
*ENPP1*	CACATCCCAGATTCCCTCACA	GCCTCAACAACTCTTCAACCCAT	XM_021087944.1	131
*β-actin*	TCTGGCACCACACCTTCT	TGATCTGGGTCATCTTCTCAC	XM_021086047.1	114

## Supplementary Materials

Supplementary materials can be found at https://www.mdpi.com/1422-0067/20/18/4549/s1.
